# LaZnB_5_O_10_, the first lanthanum zinc borate

**DOI:** 10.1107/S1600536809050922

**Published:** 2009-12-04

**Authors:** Zhi-Wei Jiao, Ru-Ji Wang, Xiao-Qing Wang, De-Zhong Shen, Guang-Qiu Shen

**Affiliations:** a Department of Chemistry, Tsinghua University, Beijing 100084, People’s Republic of China

## Abstract

Lanthanum zinc penta­borate, LaZnB_5_O_10_, was synthesized by flux-supported solid-state reaction. It is a member of the *LnM*B_5_O_10_ (*Ln *= rare earth ion and *M* = divalent metal ion) structure type. The crystal shows a three-dimensional structure constructed from two-dimensional {[B_5_O_10_]^5−^}_*n*_ layers with the lanthanum (coordination number nine) and zinc (coordination number six) ions filling in the inter­layers.

## Related literature

For general background to inorganic borates and their applications, see: Thakare *et al.* (2004 ▶); Yavetskiy *et al.* (2007 ▶); Ye & Chai (1999 ▶); Becker (1998 ▶). For related structures, see: Bernadette *et al.* (1980 ▶); Abdullaev *et al.* (1980 ▶); Campa *et al.* (1995 ▶). For the bond-valence-sum (BVS) calculation, see: Brese & O’Keeffe (1991 ▶).

## Experimental

### 

#### Crystal data

LaZnB_5_O_10_
*M* *_r_* = 418.33
Monoclinic, 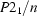

*a* = 8.7923 (19) Å
*b* = 7.629 (2) Å
*c* = 9.566 (2) Å
β = 92.667 (19)°
*V* = 641.0 (3) Å^3^
*Z* = 4
Mo *K*α radiation
μ = 10.37 mm^−1^
*T* = 295 K
0.10 × 0.08 × 0.06 mm

#### Data collection

Bruker P4 diffractometer
Absorption correction: ψ scan (North *et al.*, 1968 ▶) *T*_min_ = 0.259, *T*_max_ = 0.347
3122 measured reflections
2318 independent reflections
2174 reflections with *I* > 2σ(*I*)
*R*_int_ = 0.033
3 standard reflections every 97 reflections intensity decay: none

#### Refinement

*R*[*F* ^2^ > 2σ(*F* ^2^)] = 0.036
*wR*(*F* ^2^) = 0.086
*S* = 1.01
2318 reflections
155 parameters
Δρ_max_ = 3.81 e Å^−3^
Δρ_min_ = −1.63 e Å^−3^

### 

Data collection: *XSCANS* (Bruker, 1997 ▶); cell refinement: *XSCANS*; data reduction: *XSCANS*; program(s) used to solve structure: *SHELXS97* (Sheldrick, 2008 ▶); program(s) used to refine structure: *SHELXL97* (Sheldrick, 2008 ▶); molecular graphics: *DIAMOND* (Brandenburg, 2001 ▶); software used to prepare material for publication: *SHELXL97*.

## Supplementary Material

Crystal structure: contains datablocks I, global. DOI: 10.1107/S1600536809050922/br2126sup1.cif

Structure factors: contains datablocks I. DOI: 10.1107/S1600536809050922/br2126Isup2.hkl

Additional supplementary materials:  crystallographic information; 3D view; checkCIF report

## Figures and Tables

**Fig. 1.**
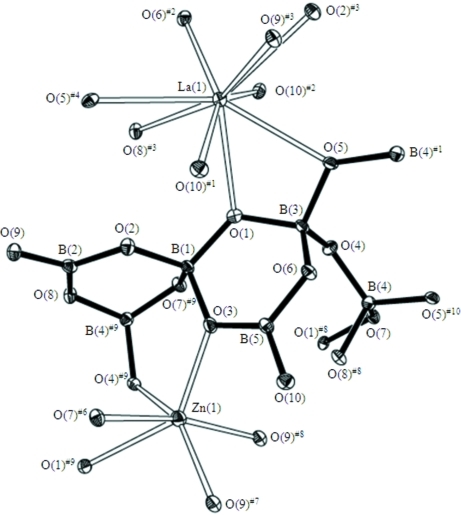
ORTEP drawing of LaZnB5O10 with 35% probability ellipsoids, showing the atomic numbering scheme.

**Fig. 2.**
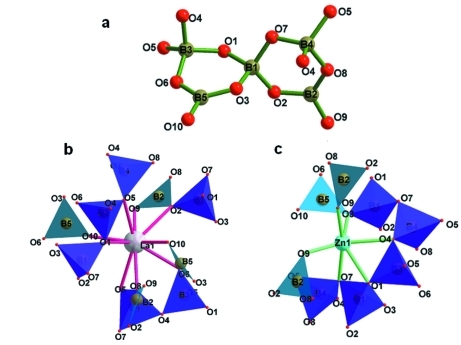
(a) The B5O12 double-ring group; (b) The coordination environment of the La atom; (c) The coordination environment of the Zn atom. The blue polyhedra are the [BO3] triangles while the purple polyhedra are the [BO4] tetrahedra in the lower two figures.

**Fig. 3.**
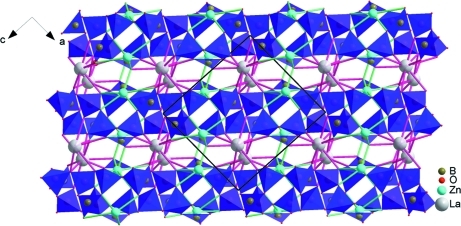
The representation of the three-dimensional LaZnB5O10 structure projected along the [010] direction with the BO3 triangles and BO4 tetrahedra. The structure contains the infinite two-dimensional [B5O10]5-n layers running almost perpendicular to the [101] direction. The La atoms are located in layers, while the Zn atoms are almost embedded in the layers.

## supplementary crystallographic information

### Comment

Inorganic borates have long been a focus of research for their wide applications as phosphors, laser materials and nonlinear optical (NLO) materials *etc* (Thakare *et al.*, 2004; Yavetskiy *et al.*, 2007; Ye *et al.*, 1999; Becker *et al.*, 1998). Among these materials, rear-earth borates especially lanthanum or yttrium borates, have been proved to be attractive matrices for lasing materials or rare-earths sensitizer-activator pairs containing phosphors. LaZnB_5_O_10_ is a new member of the family of LnMB_5_O_10_ (Ln = rear earth ions, *M* = divalent metal ions) (Abdullaev *et al.*, 1980; Bernadette *et al.*, 1980; Campa *et al.*, 1995). The asymmetric unit of LaZnB_5_O_10_ contains one unique La ion, one Zn ion, five B atoms and ten oxygen atoms as shown in Fig.1. Three BO_4_ tetrahedra and two BO_3_ triangles are linked to form a B_5_O_12_ double-ring group (Fig.2a), and these B_5_O_12_ groups are further connected to form a [B_5_O_10_]^5-^_n_ layer through sharing BO_4_ tetrahedra.

The local coordination geometries of Zn and La atoms in LaZnB_5_O_10_ are also shown in Fig.2. As can be observed, the La1 atom is bonded to nine oxygen atoms to form a distorted tetrakaidecahedron. The bond valence sum (BVS) of 3.143 for La^3+^ ions calculated by the Brese & Keeffe (Brese *et al.*, 1991) formalism shows that its valence requirement is satisfied by this coordination. The distorted tetrakaidecahedra here are further connected with each other through sharing edges to form a one-dimensional infinite chain which is arranged between the [B_5_O_10_]^5-^_n_ layers along *b* axis. The zinc cation adopts a sixfolded coordination to form a distorted octahedron. However, among these Zn—O bonds, Zn1—O1 and Zn1—O4 are significantly longer than the others. This could be probably due to the fact that the O1-O4 edge is shared with a BO_3_ group. This reduces the O1-Zn-O4 angle and tends to lengthen the bonds. Two adjacent ZnO_6_ octahedra are connected with each other through two bridging oxygen atoms and the zinc atoms are almost embedded in the [B_5_O_10_]^5-^_n_ layers. Both the zinc and lanthanum atoms link the adjacent [B_5_O_10_]^5-^_n_ layers to form a three dimensional framework (Fig.3).

### Experimental

Single crystals of the title compound were synthesized by flux-supported solid-state reaction. A mixture La_2_O_3_(99.9%), ZnO(99.0%) and H_3_BO_3_(99.99%) in the molar ratio of 1:2:14 was ground to a fine powder in a mortar and compressed into a Pt crucible. The mixture was gradually heated to 1273 K. After the mixture melted completely, it was cooled down to 1100 K at a rate of 1 °K/h, followed by cooling to room temperature at 20 °K/h. The title crystals could be obtained from the top section of the solidified melt. While in the bottom of the solidified melt, plate-like crystals were obtained which were confirmed to be LaB_3_O_6_ through the powder X-ray diffraction (PXRD) method.

### Crystal data

**Table:**

LaZnB_5_O_10_	*F*(000) = 768
*M**_r_* = 418.33	*D*_x_ = 4.335 Mg m^−^^3^
Monoclinic, *P*2_1_/*n*	Mo *K*α radiation, λ = 0.71073 Å
Hall symbol: -P 2yn	Cell parameters from 36 reflections
*a* = 8.7923 (19) Å	θ = 5.8–12.5°
*b* = 7.629 (2) Å	µ = 10.37 mm^−^^1^
*c* = 9.566 (2) Å	*T* = 295 K
β = 92.667 (19)°	Prism, colorless
*V* = 641.0 (3) Å^3^	0.10 × 0.08 × 0.06 mm
*Z* = 4	

### Data collection

**Table:**

Bruker P4 diffractometer	2174 reflections with *I* > 2σ(*I*)
Radiation source: fine-focus sealed tube	*R*_int_ = 0.033
graphite	θ_max_ = 32.5°, θ_min_ = 3.1°
ω scans	*h* = −13→1
Absorption correction: ψ scan (North *et al.*, 1968)	*k* = −1→11
*T*_min_ = 0.259, *T*_max_ = 0.347	*l* = −14→14
3122 measured reflections	3 standard reflections every 97 reflections
2318 independent reflections	intensity decay: none

### Refinement

**Table:**

Refinement on *F*^2^	Primary atom site location: structure-invariant direct methods
Least-squares matrix: full	Secondary atom site location: difference Fourier map
*R*[*F*^2^ > 2σ(*F*^2^)] = 0.036	*w* = 1/[σ^2^(*F*_o_^2^) + (0.001*P*)^2^ + 14.*P*] where *P* = (*F*_o_^2^ + 2*F*_c_^2^)/3
*wR*(*F*^2^) = 0.086	(Δ/σ)_max_ < 0.001
*S* = 1.01	Δρ_max_ = 3.81 e Å^−^^3^
2318 reflections	Δρ_min_ = −1.63 e Å^−^^3^
155 parameters	Extinction correction: *SHELXL97* (Sheldrick, 2008), Fc^*^=kFc[1+0.001xFc^2^λ^3^/sin(2θ)]^-1/4^
0 restraints	Extinction coefficient: 0.133 (3)

### Special details

**Table:**

Geometry. All e.s.d.'s (except the e.s.d. in the dihedral angle between two l.s. planes) are estimated using the full covariance matrix. The cell e.s.d.'s are taken into account individually in the estimation of e.s.d.'s in distances, angles and torsion angles; correlations between e.s.d.'s in cell parameters are only used when they are defined by crystal symmetry. An approximate (isotropic) treatment of cell e.s.d.'s is used for estimating e.s.d.'s involving l.s. planes.
Refinement. Refinement of *F*^2^ against ALL reflections. The weighted *R*-factor *wR* and goodness of fit *S* are based on *F*^2^, conventional *R*-factors *R* are based on *F*, with *F* set to zero for negative *F*^2^. The threshold expression of *F*^2^ > σ(*F*^2^) is used only for calculating *R*-factors(gt) *etc*. and is not relevant to the choice of reflections for refinement. *R*-factors based on *F*^2^ are statistically about twice as large as those based on *F*, and *R*- factors based on ALL data will be even larger.

### Fractional atomic coordinates and isotropic or equivalent isotropic displacement parameters (Å^2^)

**Table:**

	*x*	*y*	*z*	*U*_iso_*/*U*_eq_	
La1	0.18084 (3)	0.68446 (4)	0.23425 (3)	0.00861 (13)	
Zn1	0.88916 (7)	0.41215 (8)	0.38049 (7)	0.01160 (16)	
O1	0.4743 (4)	0.7209 (5)	0.2592 (4)	0.0097 (6)	
O2	0.5128 (4)	0.4196 (5)	0.1869 (4)	0.0105 (6)	
O3	0.6830 (4)	0.5372 (5)	0.3613 (4)	0.0104 (6)	
O4	0.5839 (4)	0.9780 (5)	0.3570 (4)	0.0099 (6)	
O5	0.3289 (4)	0.8921 (5)	0.4150 (4)	0.0097 (6)	
O6	0.5383 (4)	0.7202 (5)	0.5088 (4)	0.0102 (6)	
O7	0.8125 (4)	1.1547 (5)	0.3721 (4)	0.0100 (6)	
O8	0.6912 (4)	0.3737 (5)	0.0101 (4)	0.0098 (6)	
O9	0.5056 (4)	0.1522 (5)	0.0688 (4)	0.0101 (6)	
O10	0.7299 (4)	0.5391 (5)	0.6081 (4)	0.0112 (6)	
B1	0.5877 (6)	0.5824 (7)	0.2330 (5)	0.0082 (8)	
B2	0.5734 (6)	0.3144 (7)	0.0839 (6)	0.0096 (9)	
B3	0.4843 (6)	0.8275 (7)	0.3895 (6)	0.0092 (9)	
B4	0.7168 (6)	1.0340 (7)	0.4479 (5)	0.0093 (9)	
B5	0.6498 (6)	0.5943 (7)	0.4924 (6)	0.0092 (8)	

### Atomic displacement parameters (Å^2^)

**Table:**

	*U*^11^	*U*^22^	*U*^33^	*U*^12^	*U*^13^	*U*^23^
La1	0.00934 (16)	0.00822 (17)	0.00831 (16)	0.00018 (8)	0.00087 (9)	0.00037 (8)
Zn1	0.0114 (3)	0.0094 (3)	0.0140 (3)	0.0001 (2)	0.0000 (2)	−0.0002 (2)
O1	0.0091 (14)	0.0105 (15)	0.0095 (14)	−0.0007 (12)	0.0003 (11)	0.0014 (12)
O2	0.0115 (15)	0.0104 (15)	0.0098 (14)	−0.0014 (12)	0.0028 (12)	−0.0009 (12)
O3	0.0126 (15)	0.0104 (15)	0.0083 (14)	0.0029 (12)	0.0006 (11)	−0.0016 (12)
O4	0.0096 (14)	0.0099 (15)	0.0100 (14)	−0.0025 (12)	−0.0002 (11)	0.0007 (12)
O5	0.0073 (14)	0.0135 (16)	0.0085 (14)	0.0028 (12)	0.0008 (11)	−0.0006 (12)
O6	0.0107 (14)	0.0082 (14)	0.0117 (15)	0.0026 (12)	0.0010 (12)	0.0003 (12)
O7	0.0100 (15)	0.0087 (14)	0.0116 (15)	−0.0003 (12)	0.0033 (12)	−0.0006 (12)
O8	0.0108 (15)	0.0085 (15)	0.0103 (14)	−0.0028 (12)	0.0036 (12)	−0.0011 (12)
O9	0.0121 (15)	0.0087 (15)	0.0098 (15)	−0.0018 (12)	0.0018 (12)	−0.0004 (12)
O10	0.0121 (15)	0.0114 (16)	0.0099 (14)	0.0014 (13)	−0.0001 (12)	0.0020 (12)
B1	0.010 (2)	0.007 (2)	0.0076 (19)	0.0018 (16)	0.0003 (16)	0.0002 (16)
B2	0.008 (2)	0.012 (2)	0.009 (2)	0.0015 (17)	0.0019 (16)	−0.0001 (17)
B3	0.009 (2)	0.010 (2)	0.009 (2)	0.0031 (17)	0.0014 (16)	−0.0008 (16)
B4	0.010 (2)	0.010 (2)	0.0080 (19)	0.0003 (17)	0.0009 (16)	0.0005 (16)
B5	0.009 (2)	0.009 (2)	0.010 (2)	0.0006 (16)	0.0027 (16)	0.0002 (17)

### Geometric parameters (Å, °)

**Table:**

La1—O10^i^	2.385 (4)	B2—O2	1.396 (6)
La1—O10^ii^	2.478 (4)	B1—O2	1.464 (7)
La1—O6^ii^	2.549 (4)	B5—O3	1.372 (6)
La1—O9^iii^	2.566 (4)	B1—O3	1.495 (6)
La1—O1	2.595 (4)	B3—O4	1.486 (7)
La1—O2^iii^	2.608 (4)	B4—O4	1.486 (7)
La1—O5	2.643 (4)	B3—O5	1.484 (6)
La1—O5^iv^	2.648 (4)	B4—O5^x^	1.500 (6)
La1—O8^v^	2.678 (4)	B5—O6	1.387 (6)
Zn1—O3	2.049 (4)	B3—O6	1.466 (7)
Zn1—O7^vi^	2.077 (4)	B4—O7	1.462 (7)
Zn1—O9^vii^	2.088 (4)	B1—O7^ix^	1.472 (6)
Zn1—O9^viii^	2.099 (4)	B2—O8	1.358 (6)
Zn1—O1^ix^	2.346 (4)	B4—O8^viii^	1.511 (7)
Zn1—O4^ix^	2.350 (4)	B2—O9	1.378 (7)
B1—O1	1.482 (6)	B5—O10	1.351 (6)
B3—O1	1.488 (7)		
			
O10^i^—La1—O10^ii^	148.99 (6)	O7^vi^—Zn1—O9^vii^	87.47 (15)
O10^i^—La1—O6^ii^	150.84 (13)	O3—Zn1—O9^viii^	89.60 (15)
O10^ii^—La1—O6^ii^	55.60 (12)	O7^vi^—Zn1—O9^viii^	166.66 (15)
O10^i^—La1—O9^iii^	70.71 (13)	O9^vii^—Zn1—O9^viii^	79.20 (16)
O10^ii^—La1—O9^iii^	124.81 (12)	O3—Zn1—O1^ix^	135.44 (14)
O6^ii^—La1—O9^iii^	110.05 (12)	O7^vi^—Zn1—O1^ix^	64.12 (14)
O10^i^—La1—O1	73.86 (13)	O9^vii^—Zn1—O1^ix^	95.83 (14)
O10^ii^—La1—O1	76.04 (12)	O9^viii^—Zn1—O1^ix^	116.27 (14)
O6^ii^—La1—O1	119.66 (12)	O3—Zn1—O4^ix^	86.69 (14)
O9^iii^—La1—O1	127.52 (12)	O7^vi^—Zn1—O4^ix^	102.25 (14)
O10^i^—La1—O2^iii^	120.59 (12)	O9^vii^—Zn1—O4^ix^	144.77 (14)
O10^ii^—La1—O2^iii^	71.71 (12)	O9^viii^—Zn1—O4^ix^	88.49 (14)
O6^ii^—La1—O2^iii^	75.30 (12)	O1^ix^—Zn1—O4^ix^	60.35 (13)
O9^iii^—La1—O2^iii^	53.55 (12)	O2—B1—O7^ix^	112.7 (4)
O1—La1—O2^iii^	124.01 (12)	O2—B1—O1	111.0 (4)
O10^i^—La1—O5	82.96 (13)	O7^ix^—B1—O1	106.0 (4)
O10^ii^—La1—O5	73.55 (12)	O2—B1—O3	106.2 (4)
O6^ii^—La1—O5	126.16 (12)	O7^ix^—B1—O3	108.5 (4)
O9^iii^—La1—O5	83.62 (12)	O1—B1—O3	112.4 (4)
O1—La1—O5	54.44 (12)	O8—B2—O9	125.6 (5)
O2^iii^—La1—O5	72.95 (12)	O8—B2—O2	120.1 (5)
O10^i^—La1—O5^iv^	74.93 (12)	O9—B2—O2	114.4 (4)
O10^ii^—La1—O5^iv^	117.16 (12)	O6—B3—O5	108.9 (4)
O6^ii^—La1—O5^iv^	77.42 (12)	O6—B3—O4	115.0 (4)
O9^iii^—La1—O5^iv^	108.04 (12)	O5—B3—O4	109.5 (4)
O1—La1—O5^iv^	98.43 (12)	O6—B3—O1	110.6 (4)
O2^iii^—La1—O5^iv^	136.90 (12)	O5—B3—O1	107.5 (4)
O5—La1—O5^iv^	149.36 (8)	O4—B3—O1	105.1 (4)
O10^i^—La1—O8^v^	107.11 (12)	O7—B4—O4	110.2 (4)
O10^ii^—La1—O8^v^	68.05 (12)	O7—B4—O5^x^	112.4 (4)
O6^ii^—La1—O8^v^	61.25 (12)	O4—B4—O5^x^	112.6 (4)
O9^iii^—La1—O8^v^	159.02 (12)	O7—B4—O8^viii^	109.2 (4)
O1—La1—O8^v^	69.00 (12)	O4—B4—O8^viii^	108.6 (4)
O2^iii^—La1—O8^v^	132.27 (12)	O5^x^—B4—O8^viii^	103.5 (4)
O5—La1—O8^v^	117.15 (11)	O10—B5—O3	121.6 (5)
O5^iv^—La1—O8^v^	52.70 (11)	O10—B5—O6	117.9 (4)
O3—Zn1—O7^vi^	98.79 (16)	O3—B5—O6	120.4 (5)
O3—Zn1—O9^vii^	125.64 (15)		

Symmetry codes: (i) −*x*+1, −*y*+1, −*z*+1; (ii) *x*−1/2, −*y*+3/2, *z*−1/2; (iii) −*x*+1/2, *y*+1/2, −*z*+1/2; (iv) −*x*+1/2, *y*−1/2, −*z*+1/2; (v) −*x*+1, −*y*+1, −*z*; (vi) *x*, *y*−1, *z*; (vii) *x*+1/2, −*y*+1/2, *z*+1/2; (viii) −*x*+3/2, *y*+1/2, −*z*+1/2; (ix) −*x*+3/2, *y*−1/2, −*z*+1/2; (x) −*x*+1, −*y*+2, −*z*+1.
